# Analysis of Matched Tumor and Normal Profiles Reveals Common Transcriptional and Epigenetic Signals Shared across Cancer Types

**DOI:** 10.1371/journal.pone.0142618

**Published:** 2015-11-10

**Authors:** Andrew M. Gross, Jason F. Kreisberg, Trey Ideker

**Affiliations:** 1 Bioinformatics and Systems Biology Program, University of California San Diego, La Jolla, California, United States of America; 2 Department of Medicine, University of California San Diego, La Jolla, California, United States of America; Northwestern University, UNITED STATES

## Abstract

To identify the transcriptional regulatory changes that are most widespread in solid tumors, we performed a pan-cancer analysis using over 600 pairs of tumors and adjacent normal tissues profiled in The Cancer Genome Atlas (TCGA). Frequency of upregulation was calculated across mRNA expression levels, microRNA expression levels and CpG methylation sites and is provided here as a resource. Frequent tumor-associated alterations were identified using a simple statistical approach. Many of the identified changes were consistent with the increased rate of cell division in cancer, such as the overexpression of cell cycle genes and hypermethylation of PRC2 binding sites. However, we also identified proliferation-independent alterations, which highlight novel pathways essential to tumor formation. Nearly all of the GABA receptors are frequently downregulated, with the gene encoding the delta subunit (GABRD) strongly upregulated as the notable exception. Metabolic genes are also frequently downregulated, particularly alcohol dehydrogenases and others consistent with the decreased role of oxidative phosphorylation in cancerous cells. Alterations in the composition of GABA receptors and metabolism may play a key role in the differentiation of cancer cells, independent of proliferation.

## Introduction

Cancerous cells are characterized by numerous changes to the genome, epigenome, transcriptome. While most tumor-associated changes have little function, key genes and pathways are often implicated by looking across patients within a cohort for events that are recurrent [1–3]. While such analyses are traditionally performed across well-defined patient populations with tumors of similar anatomical location and histological appearance, large data sets produced by public efforts such as The Cancer Genome Atlas (TCGA) [2, 4] have now made meta-analysis of cancer studies feasible.

By looking across many different subtypes, pan-cancer analyses provide a high level, tissue agnostic view of cancer. Many such studies have analyzed coordinated changes across molecular phenotypes and clinical data to isolate key signals during tumorgenesis. Such efforts have uncovered conserved patterns of gene co-expression across many types of tumors [5, 6] identifying molecular patterns associated with tumor growth and proliferation. In a complementary approach, a recent paper by Gentles and colleagues [7] identified genes whose expression was associated with survival across cohorts spanning many tissues. These authors found that the overexpression of genes near the FOXM1 transcriptional network and of genes that drive cell cycle progression were associated with adverse patient outcomes. These highly conserved signatures of cell proliferation support the hypothesis that a core cancer phenotype is activated to varying degrees across diverse tumor types.

Thus far, such pan-cancer studies of transcriptional changes have focused mainly on tumor samples, without consideration of normal tissue. In contrast, studies of mutations, structural variations or DNA copy number alterations have frequently relied on subtractive analysis of matched data to achieve power in detecting tumor-specific changes. Although a few expression studies analyzed patient-matched tumors and adjacent normal tissue, these studies were restricted to specific tissue cohorts [8–13]. They were thus capable of identifying genes whose expression in tumor deviates from normal in a single tissue, but were unable to distinguish which of these changes are specific to a given study population or are general features of cancer as a whole. To this effect, a pan-cancer analysis of differential transcriptional regulatory programs—whether at the level of mRNA expression, miRNA expression or methylation—has not yet been performed.

Here, we perform such an analysis using information readily available in The Cancer Genome Atlas (TCGA), which has enabled standard data collection procedures and molecular profiling assays for numerous measurement platforms [4]. Using TCGA data, we compile a comprehensive list of tumor-associated mRNAs, miRNAs and methylation sites by measuring the frequency at which their levels are elevated between matched tumor and normal samples across all measured cancer tissues. The upregulation frequencies for these features are provided as a general resource to the cancer community. We find that in addition to near-universal overexpression of genes important for tumor proliferation, there exist prominent proliferation-independent signals which could play a role in tissue remodeling.

## Results

To identify ubiquitous tumor-associated signals, we downloaded all of the available data from TCGA as of April 2, 2015, through the Broad Institute’s Firehose web portal (Methods) [14]. This dataset consisted of genome-wide mRNA expression, microRNA (miRNA) expression and CpG methylation for over 9,000 tumors, of which adjacent normal tissues were also profiled for over 600 patients (S1 Fig).

**Fig 1:**
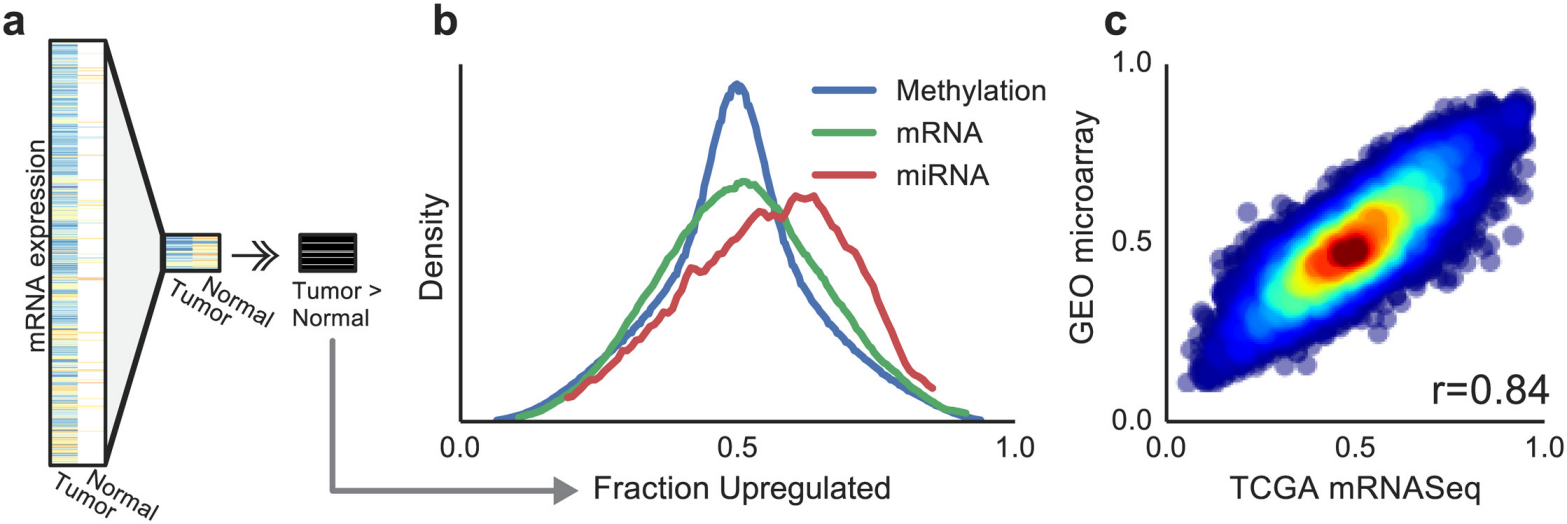
Description of the *f*_*up*_ statistic. (a) Schematic of the calculation of fraction upregulated (*f*_*up*_) for a single gene expression profile across the TCGA cohort. Data are filtered to include only matched samples, the magnitudes of paired tumor/normal samples are compared, and a fraction of how often the gene is upregulated is recorded. (b) Density of *f*_*up*_ statistic across genome-wide mRNA, miRNA, and methylation measurements. (c) Comparison of mRNA *f*_*up*_ statistic calculated from TCGA mRNASeq measurements versus microarray measurements downloaded from GEO.

Given this large collection of matched tumor and normal data, we were powered to employ a simplified analysis to identify molecular signals associated with tumors (Methods, Fig 1a and S2 Fig). For each mRNA, miRNA or CpG marker, we quantified fraction upregulated (*f*_*up*_), the fraction of patients for which the marker level was higher in the tumor than in the matched normal tissue. This metric is a formulation of the sign-test statistic *p* = Pr(**x**_**i**_ > **y**_**i**_), where **x** and **y** are vectors of matched samples from tumor and adjacent normal tissue, respectively. Using this statistic we identified mRNAs, miRNAs and CpGs that ranged from random (*f*_*up*_ = 0.5) to highly differentially expressed or methylated (*f*_*up*_ approaching 0 or 1) (Fig 1b and S1 Table). To assess the reproducibility of this statistic, we studied 10 additional gene expression microarray datasets, spanning 1012 subjects with matched tumor/normal data from the Gene Expression Omnibus. After calculating *f*_*up*_ for all of the genes in the dataset, we found a correlation of 0.84 (*P* < 10^−16^, 95% confidence interval (CI): 0.838–0.847) between these scores and the *f*_*up*_ scores identified from TCGA RNA-sequencing data (Fig 1c and S2 Table).

**Fig 2:**
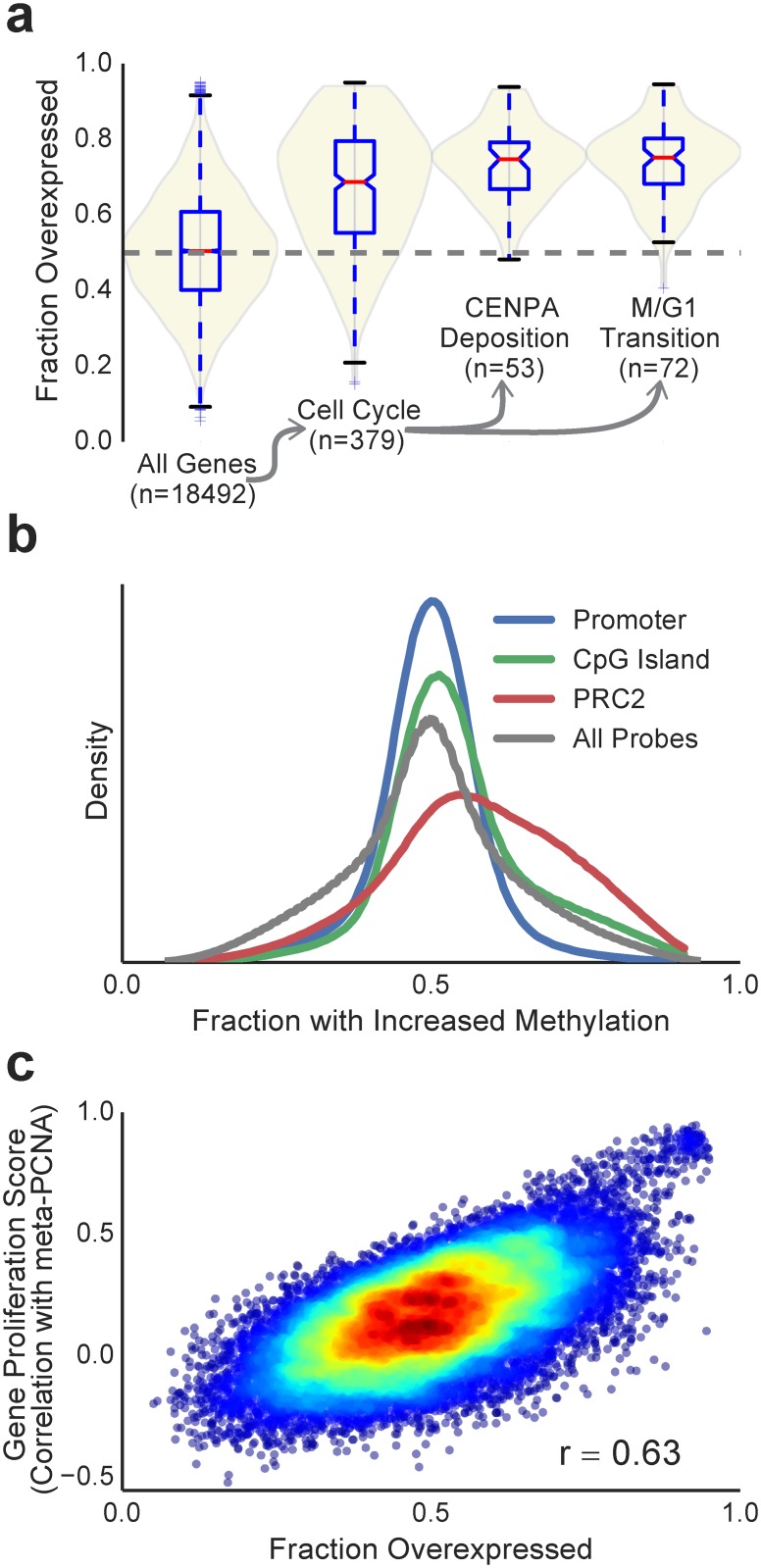
Tumor-associated features are consistent with proliferative signals. (a) Violin plots showing distribution of mRNA level *f*_*up*_ statistic (fraction overexpressed) across all genes, compared to genes annotated to the cell cycle and its subsets: “deposition of CENPA containing nucleosomes at the centromere” and “M/G1 transition” in mSigDB. (b) Density plots of the distribution of *f*_*up*_ (fraction with increased methylation) across methylation markers annotated to functional genomic sites. (c) Scatter plot comparing *f*_*up*_ statistic against gene correlation with proliferation for every gene expression profile.

Inspection of molecular entities with extreme values of *f*_*up*_ confirmed that tumor proliferation plays a dominant role, as described by previous studies [5–7, 15–16]. Among the most heavily tumor-associated genes was FOXM1, for which the mRNA levels are upregulated in 93% of patient tumors (95% CI_Bonf_: 87%-97%). FOXM1 is a well-known proliferation-associated transcription factor which plays a central role in regulating the progression of the cell cycle [16]. Gene-Set Enrichment Analysis highlighted a number of features associated with proliferation, including upregulation of cell cycle genes with particularly large effect sizes observed for the cell cycle gene subsets “deposition of CENPA containing nucleosomes at the centromere” and “M/G1 transition” (Fig 2a and S3 Table, Mann-Whitney U test, *P*_*BH*_ < 10^−16^). Analysis of methylation markers showed hypermethylation occurring at PRC2 binding sites which have been previously linked to proliferation in cancer [17] (Fig 2b). Taken together, these findings confirm that many tumor-associated molecular changes are driven by proliferation.

To isolate proliferation dependent and independent components of the tumor associated signal, we assigned a proliferation score for each mRNA, miRNA and methylation site. This was calculated by assessing the correlation across TCGA patients of each feature expression level with a previously published proliferation signature [18] (meta-PCNA, Methods). Indeed we found that these proliferation scores were highly correlated with *f*_*up*_ scores across all three data types, with Pearson’s *r* = 0.63 (95% CI: 0.62–0.64), 0.62 (0.56–0.67), and 0.674 (0.672–0.676) for mRNA, miRNA and methylation, respectively (Fig 2c, for all three statistics *P* < 10^−16^). Interestingly, we observed a heavy skew in the *f*_*up*_ statistic for miRNA species in particular (Fig 1a), which we attribute to a general trend of increasing miRNA expression with proliferation [19].

To assess tumor-associated, growth-independent signals, we adjusted marker levels to remove any association with proliferation and recalculated *f*_*up*_ (i.e., accounting for the meta-PCNA signature, see Methods, S4 Table). We expected that features with extreme values of detrended *f*_*up*_ would be altered in the transition from normal to tumor cells, but not associated the tumor growth rate. Enrichment analysis of this detrended statistic identified overexpression of genes involved in ribosomal and proteasomal processes (S5 Table, Mann-Whitney U test, *P*_*BH*_ < 10^−16^, *P*_*BH*_ < 10^−7^, respectively). Interestingly, while telomere maintenance genes had a general increase in *f*_*up*_, genes involved with telomere extension had much stronger correlations with proliferation than genes involved in packaging of telomere ends (*P* < 0.001, S3 Fig). It is likely that these and other pathways are important for the initial rewiring of the cell required for accelerated growth but then have little impact on the tumor’s growth rate.

**Fig 3:**
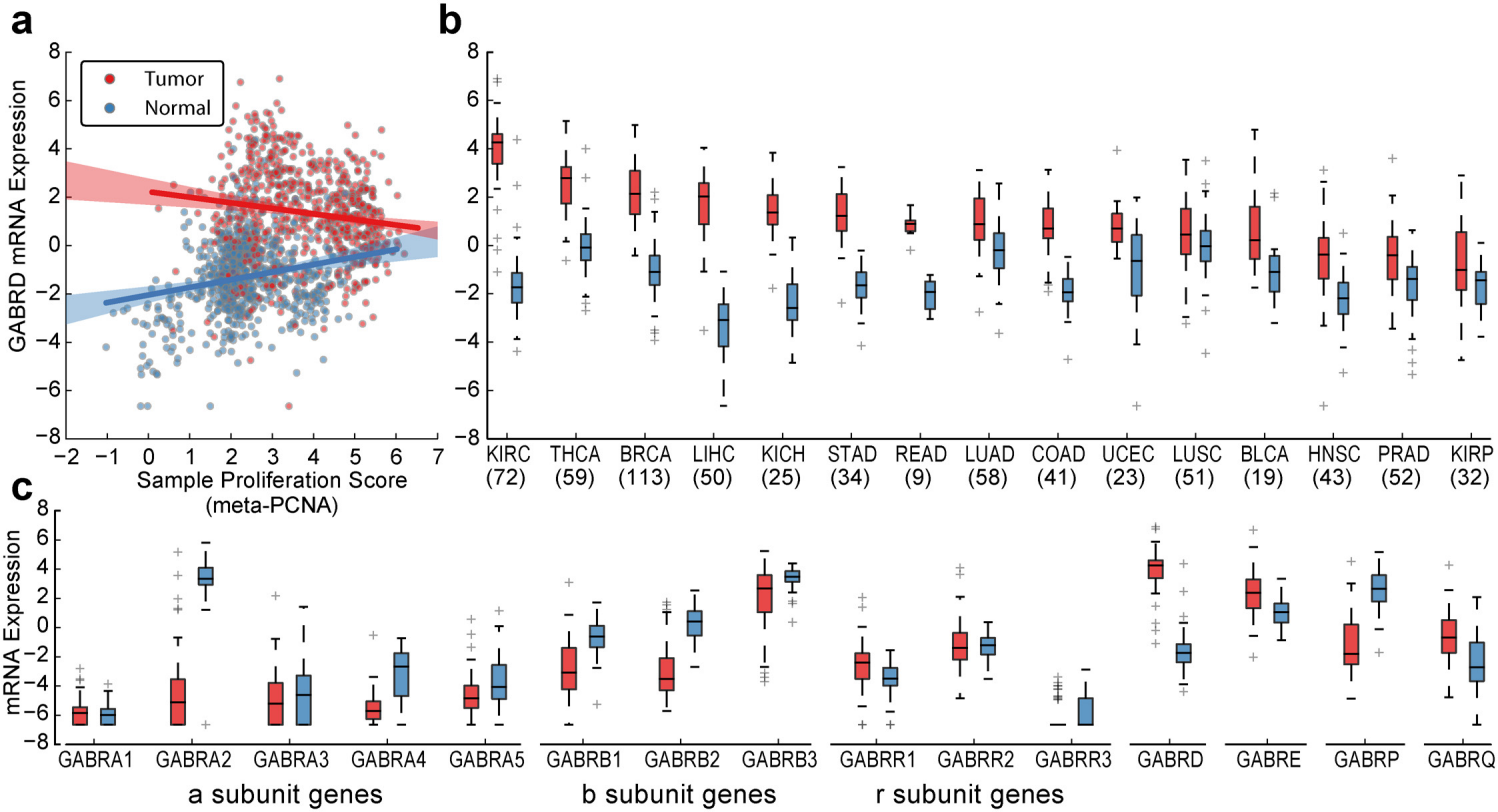
GABRD is tumor-associated, independent of proliferation. (a) Scatter-plot comparing GABRD gene expression profiles to proliferation scores across matched tumor and normal samples. Lines indicate linear regression figs of tumor (red) and normal (blue) samples, shaded regions indicate 95% confidence intervals. (b) Comparison of matched tumor and normal profiles for GABRD expression, grouped by tissue type. (c) Comparison of matched tumor and normal profiles for all GABA protein subunits in renal cell carcinoma. Cancer acronyms are defined as follows: KIRC, kidney renal clear cell carcinoma; THCA, thyroid carcinoma; BRCA, breast invasive carcinoma; LIHC, liver hepatocellular carcinoma; KICH, kidney chromophobe; STAD, stomach adenocarcinoma; READ, rectum adenocarcinoma; LUAD, lung adenocarcinoma; COAD, colon adenocarcinoma; UCEC, uterine corpus endometrioid carcinoma; LUSC, lung squamous cell carcinoma; BLCA, bladder urothelial carcinoma; HNSC, head and neck squamous cell carcinoma; PRAD, prostate adenocarcinoma; KIRP, kidney renal papillary cell carcinoma.

**Fig 4:**
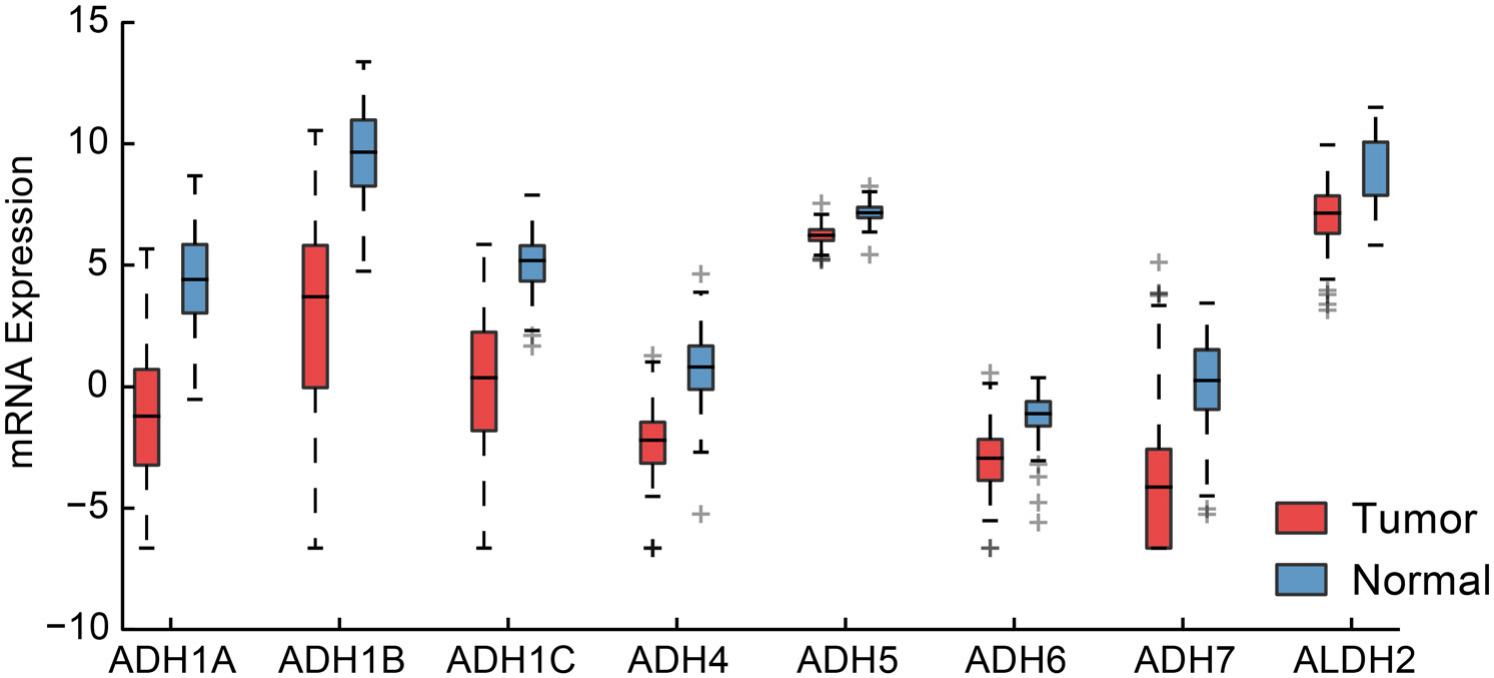
Differential expression of alcohol dehydrogenase family of genes. Shown here for TCGA breast cancer dataset as a representative cohort. Also shown is ALDH2 which is the major enzyme responsible for breaking down acetaldehyde, the primary intermediate product of alcohol metabolism.

The most upregulated, proliferation-independent genes in tumors were SEMA5B (detrended *f*_*up*_ = 0.82 [0.74–0.88], S4 Fig), the GABA receptor subunit GABRD (detrended *f*_*up*_ = 0.82 [0.64–0.80], Fig 3), and the well-studied tumor suppressor CDKN2A (detrended *f*_*up*_ = 0.72 [0.63–0.79]). SEMA5B is a gene in the semaphorin family, whose main roles are to serve as guidance signals in various stages of development. These genes have recently been shown have a role in cancer signaling [20]. This GABA_A_ subunit is primarily expressed in the cerebellum where its receptor is located extrasynaptically [21–22], but it is also expressed in the testes (S5 Fig) and CD4+ T-cells [22–23]. In the TCGA dataset, GABRD is overexpressed in 89% (CI_Bonf_ 81%-93%) of subjects and has a slight negative association with proliferation in tumors (Fig 3). In contrast, most other GABA subunit genes are downregulated across many cancers (Fig 3c, S6 Fig). We observed a particularly large effect in renal cell carcinoma where there is a ten-fold median decrease in GABRA2 alongside a six-fold increase in expression of GABRD (Fig 4e). Similar effects were observed in a paired microarray dataset (S7 Fig).

Gene sets with similar patterns of differential expression as GABRD included ‘hematopoietic cell lineage’ and ‘‘helper T-cell polarization’ (Methods). Further inspection of genes in the helper T-cell polarization pathway showed a preference for genes expressed in Th1 as opposed to Th2 cells. To determine whether this signal represented infiltration by immune cells into the tumor, we used the CIBERSORT program [7] to predict immune cell subsets in tumor samples, but found little to no association with GABRD. While it remains difficult to completely rule out immune infiltration as a driving force of this signal, these findings suggest that increased levels of the delta subunit could lead to functional changes in the GABA_A_ receptor that may play a role in tumor cell differentiation.

Among the most downregulated, proliferation-independent genes we noticed widespread epigenetic silencing in tumors with strong enrichments for transcription start site hypermethylation (Methods, S8a Fig, Odds-Ratio = 2, *P* < 10^−16^) and gene body hypomethylation (S8b Fig, Odds-ratio = 2.5, *P* < 10^−16^). While coverage of methylation markers on the Illumina 450k chip varied across genes, manual inspection (Methods) of the most consistently downregulated genes identified many genes with associated with methylation changes to their DNA including GSTM5 (detrended *f*_*up*_ = 0.27 [0.19–0.35], S8c Fig) and NRXN1 (detrended *f*_*up*_ = 0.25 [0.18–0.34], S8d Fig). While NRXN1 is primarily expressed in brain where it serves as a cell surface protein, it has also been shown to play a role in remodeling of vascular tissue indicating it may play a wider role in regulation of cell adhesion in the periphery [24].

A screen for gene-sets enriched for proliferation-independent downregulation identified transcription and fatty acid metabolism pathways (Mann-Whitney U test, *P*_*BH*_ < 10^−8^, *P*_*BH*_ < 10^−4^, respectively). Among the fatty acid metabolism gene set were the alcohol dehydrogenase genes which were nearly ubiquitously down-regulated with a particularly large effect for the class I genes (*f*_*up*_ = 0.06 [0.02–0.10], 0.05 [0.02–0.10] and 0.12 [0.06–0.18] for ADH1-A, -B and -C, respectively) as well as ALDH2 (*f*_*up*_ = 0.15 [0.09–0.22]), which serves to break down acetaldehyde (Fig 4 and S9 Fig). The downregulation of alcohol metabolism is likely a component of alternative pyruvate usage mediated by the Warburg effect in which cancer cells increase their rate of glycolysis by shifting to aerobic metabolism [25]. Exploration of other glycolysis genes supported this shift with upregulation of the lactate dehydrogenase gene LDHA (*f*_*up*_ = 0.79 [0.71–0.86]) alongside downregulation of the mitochondrial pyruvate carrier gene MPC1 (*f*_*up*_ = 0.11 [0.09–0.22], TCGA symbol BRP44L). Much like the ADH genes, MPC1 is downregulated in a proliferation-independent manner, and has recently been shown to affect cancer cell line growth in nonadherent, 3D culture conditions but not in proliferation or cell-cycle progression assays [26].

## Discussion

Here we have provided a resource to aid in the understanding of tumor-associated molecular changes. Using the largest database of molecular profiles from paired tumor and adjacent normal tissues available, we determined how often each mRNA, miRNA and methylation site is differentially expressed in cancer.

We observed changes in the expression levels of features associated with growth and proliferation, including cell cycle genes, global miRNA expression and methylation of PRC2 binding sites. In addition to features consistent with rapid cellular proliferation, we also observed a number of proliferation-independent signals. These genes may lie in pathways required for cells to break free of the normal mechanisms that regulate properties such as telomere processing and tissue invasiveness. Such a proliferation-independent pattern could also arise for tumor suppressors. Many tumor suppressors are activated in response to DNA damage but may be actively suppressed by altered molecular signaling in tumors.

One major finding of this study is the proliferation-independent upregulation of GABRD in nearly all tumors profiled. In addition to its well-known role of neurological signaling, signaling via GABA subunits can also suppress the proliferation of both neural and peripheral stem cells. In addition, dysregulation of GABA signaling has been implicated in various cancers, where it is hypothesised to have a role in the differentiation and proliferation of tumor stem cells [27].

There are a number of possible explanations for why many GABA subunits are downregulated, but GABRD in particular is upregulated, in cancer. One possibility is that tumors express a novel receptor configuration; another is that the expression of the delta subunit could create non-functional receptors with other subunits. While it is hard to rule out the former explanation, the expression of GABRD in the testes (S5 Fig), and the observation that GABA has been shown to promote proliferation of Leydig cells in rodent testes [28], gives some weight to the idea that usage of an alternative GABA_A_ receptor may be important for tumorigenesis.

Further work is clearly needed to understand the proliferation-independent genes and expand on their role in cancer. While secondary validation methods often measure the change of a cell line’s growth rate in response to disruption of a target, phenotypes such as those described here would not likely manifest in such assays. In contrast, non-traditional assays such as cell migration and 3D cell culture may be required to validate such phenotypes. 3D cell culture experiments have recently been conducted on the pyruvate carrier MPC1 in which the coauthors show a clear induction of growth only when this gene is re-expressed in 3D culture and mouse xenograft models, not in classical (2D) cell culture [26].

Finally we would like to highlight the utility of using a large, diverse cohort to derive a robust pan-cancer signal. It is important to note that we do not aim to diminish the importance that normal tissue function, exposure to carcinogens, and cell turnover rates can have on the phenotypes of different cancer presentations. However, signals that are robust to tissue and environmental context are likely to be very important to the core processes driving a broad spectrum of cancer types. With the recent attention towards precision medicine, it is all the more important to define the standard molecular phenotype for cancer in general: Only by first defining common molecular features can we truly understand how treatment can be catered to detect and attack specific presentations of the disease.

## Methods

### Informed Consent

Informed consent was obtained for all patients as part of the Cancer Genome Atlas consortia. All data used in this study were downloaded from public websites after the data were consented for public use. No handling of personally identifiable information was done by the researchers on this study.

### Molecular Data Retrieval and Processing

All data were downloaded using the Broad Institute’s firehose_get data-retrieval utility. To maintain the coherency of the analysis across different data layers and cancer types, we used Level 3 normalized molecular data as the input to our analysis and used all data available as of the April 2, 2015 standard data run. The use of the TCGA Genome Data Analysis Center (GDAC) pipeline is intended to make these results easy to update as more TCGA data become available.

For TCGA gene expression values, we used data provided by Rahman and colleagues, who reprocessed the RNA sequence based expression data and showed better performance on controls [29]. While using this data as opposed the standard TCGA pipeline yielded slight changes to the results presented here, they are qualitatively very similar for both pipelines. To maintain consistency and respect data versioning we only used patients and genes present in the Firehose dataset.

A marker (gene, miRNA, methylation probe) filter was applied to TCGA data to ensure that there was a detectable change in value between patient matched tumor and normal profiles in at least 50% of subjects. In general, this approach removed features whose levels were below the limit of detection in both tumor and normal, resulting in identical low values. The resulting feature set consisted of 396,059 methylation probes, 520 microRNA, and 18420 genes.

Microarray data was retrieved via manual search of the Gene Expression Omnibus (GEO) for large molecular cohorts with paired tumor/normal expression data from the following accessions: GSE25097, GSE14520, GSE62872, GSE44076, GSE53757, GSE39791, GSE5364, GSE41258, GSE39004, GSE68468 and GSE33532. Data were obtained from the pre-processed series matrix files made available on GEO, and probes were averaged onto their annotated genes. Due to the unbalanced distribution of tissues available on GEO, fraction upregulated (*f*_*up*_) statistics were calculated for each tissue type individually, and then averaged to obtain a consensus. As not all microarray platforms had full coverage of the coding genes, statistics were calculated for available data, and genes profiled in fewer than 500 matched samples were discarded. This resulted in 16785 genes for which both microarray and RNA-sequencing data were available.

### Assessment of Differential Expression via the Fraction of Upregulated Patients

The fraction upregulated metric is a formulation of the sign-test statistic *p* = Pr(**x**_**i**_ > **y**_**i**_), where **x** and **y** are vectors of matched samples. This statistic can be seen as a simplification of the Wilcoxon signed rank test, as it does not use the magnitude of the differences for a ranking but rather counts the signs of the differences. This is a simple, assumption-free metric in which information on the magnitude of differential expression or methylation is discarded. The statistic represents the fraction of patients for which a marker takes on a higher value in the tumor than the matched normal sample and ranges between 0 and 1. Statistical assessment of *f*_*up*_ is conducted by testing against the null hypothesis that *f*_*up*_ assumes a binomial distribution with a mean of 0.5. Confidence intervals are assessed via examination of a beta distribution fit with shape parameters defined by the sign test. Although such a procedure can greatly limit statistical power when the sample size is small, at large sample sizes, *f*_*up*_ tracks very well with parametric statistics such as a paired t-test (S2 Fig).

By simplifying to a sign test we lose statistical power, but gain robustness of the test by allowing for application of this test regardless of the distribution of the data. This is used in replacement of standard statistical techniques used such as a paired t-test or specialized differential expression tools which pool variance across markers that are traditionally used in studies that have much smaller sample sizes (generally *n* = 3–20) and thus lack the power to use such a simplified model. We refrain from using such techniques as they would introduce a wide variety of confounding factors which would make our analysis much less robust and harder for the reader to interpret. For example the use of a t-test without modeling tumor purity as a covariate would be inappropriate in this setting as more pure samples would have an outsized effect.

Furthermore this nonparametric exact test has a number of desirable properties for integrative analysis across datasets. Statistically it relies on no assumptions and is robust to outliers. Furthermore it does not pool samples as biological replicates and thus gives all samples equal weights when calculating a summary value. Biologically the sole assumption of the test is that the tumor sample contains more tumor cells than the normal sample. Due to these properties, we expect little contribution of non-cancer tissue-specific expression and batch effects.

### Proliferation Scoring

A patient level proliferation score was adopted from the meta-PCNA metric published in Venet *et al*. [18]. This previous study mined normal, non-diseased tissues and defined a set of 131 genes associated with the well-studied Proliferating Cell Nuclear Antigen (PCNA) gene, then created a meta-gene calculated as the median expression level of these 131 genes. As in Venet *et al*., the median of these genes was used to construct the proliferation score in the current study. A marker-level association with this proliferation score was then computed for each gene, miRNA or methylation probe by assessing the Pearson correlation of the change in meta-PCNA with the change in marker levels from tumor to normal tissue for all subjects with matched samples.

### Assessment of Proliferation-Independent Tumor-Associated Features

To search for features that are tumor-associated independently of proliferation, the association of marker levels with proliferation (meta-PCNA) was detrended via a linear model. The detrended *f*_*up*_ metric is very similar to the standard *f*_*up*_ calculation with the addition of preprocessing to remove the trends of proliferation. Additional tissue and interaction terms are added to model to association of metaPCNA with tissue.

The detrending step is implemented in R using the following model:

marker_level ~ metaPCNA + tissue + metaPCNA: tissue

Where metaPCNA:tissue is an interaction term between these two factors. After this model is fit for all markers we obtain a matrix of residuals from the set of markers, and repeat the screen for conserved changes as previously implemented for *f*_*up*_. The screen result provides us with p-values and confidence intervals for all detrended *f*_*up*_ values.

### Gene Set Enrichment Analysis

Gene sets were downloaded from the Molecular Signatures Database (mSigDB) [30]. Version 5 of the canonical pathway gene sets was used in this analysis. Enrichment of *f*_*up*_ for gene sets was performed by screening all sets for a difference in the distribution of *f*_*up*_ within the set as compared to the background gene set via the rank-based Mann-Whitney U test.

To understand whether GABRD had coordinated differential expression with any annotated pathways, we conducted an enrichment test against the co-differential expression of GABRD with all other genes. To address this, we assessed enrichment of co-differential expression by the following method:

- dx: gene x gene correlation across matrix of differential expression
- dt: gene x gene correlation across matrix of tumor-only gene expression
- cx: dx—dt, change in correlation
- pathway enrichment: change in mean of cx within genes annotated to a given pathway

During preliminary analysis we noted that proliferation associated pathways were enriched for co-differential expression with many genes. We suspect this is the case due to the strong proliferation component of the differential expression signal giving these genes more information content. To hone in on pathways with a specific enrichment for GABRD we computed pathway enrichments for all genes, and ranked GABRD with respect to all other genes. For the two pathways highlighted in the text, ‘hematopoietic cell lineage’ and ‘helper T-cell polarization’ the enrichment of GABRD was ranked 3rd and 9th of all genes profiled.

mSigDB pathway IDs for gene sets cited in the main text are as follows:

- cell cycle: M5336
- deposition of CENPAcontaining nucleosomes at the centromere: M871
- M/G1 transition: M10080
- hematopoietic cell lineage: M6856
- helper T-cell polarization: M4047
- ribosome: M189
- proteasome: M10680
- packing of telomere ends: M17695
- telomere extension: M14804
- telomere maintenance: M4052

### Integration of Methylation and Expression Data-Layers

To understand epigenetic silencing of frequently downregulated genes, we integrated data from the DNA methylation and gene expression data-layers. This analysis took place on the 357 patients with both data-types profiled across tumor and normal tissue samples. Genes were annotated as up- or down-regulated by the significance of the detrended *f*_*up*_ metric with a threshold of *P*_*Bonf*_ < 0.05. The odds-ratio statistic in the main text was constructed by comparing the frequency at which methylation probes were greater or less than the median value of the distribution for probes mapping to downregulated genes against all other probes. To further explore epigenetic silencing, we manually inspected the 10 most proliferation-independent downregulated genes. While multiple-hypothesis corrected p-values of associations are not reported in S8 Fig, we estimate test space to be on the order of 100 tests as 10 genes were explored and around 10 possible combinations of annotations could be constructed.

### Availability

All data retrieval and processing steps are documented in a series of IPython notebooks [31] available online (https://github.com/theandygross/TCGA_differential_expression). These notebooks provide fully executable instructions for the reproduction of the analyses and the generation of figures and statistics for this study.

## Supporting Information

S1 Fig Sample counts of TCGA patients with matched tumor/normal data. (PDF)

S2 Fig Comparison of the *f*_*up*_ up/down statistic to the paired t-test as an alternative metric. Shown for all genes across the pan-cancer TCGA mRNA sequencing cohort.
(PDF)

S3 Fig Scatter plot comparing gene-level proliferation score against fraction upregulated for genes involved in telomere end packaging and telomere extension. (PDF)

S4 Fig SEMA5B is tumor-associated, independent of proliferation. (a) Scatter-plot comparing SEMA5B gene expression profiles to proliferation scores across matched tumor and normal samples. Lines indicate linear regression figs of tumor (red) and normal (blue) samples, shaded regions indicate 95% confidence intervals. (b) Comparison of matched tumor and normal profiles for SEMA5B expression, grouped by tissue type. (c) Comparison of matched tumor and normal profiles for all SEMA protein family of genes in renal cell carcinoma (note that the x-tick labels correspond to the gene suffix, e.g. 3A represents SEMA3A).
(PDF)

S5 Fig Violin plot of of GABA_A_ subunit gene expression in the testis. Data obtained from the Genotype-Tissue Expression (GTEX) project [22].
(PDF)

S6 Fig Paired tumor-normal expression for GABA receptor genes across different tissues. (PDF)

S7 Fig Characterization of GABRD in a paired microarray dataset. (a) Scatter plot comparing GABRD gene expression profiles to proliferation scores across matched tumor and normal samples. (b) Comparison of matched tumor and normal profiles for all GABA protein subunits.
(PDF)

S8 Fig Exploration of epigenetic silencing in consistently downregulated genes. (a-b) Distribution of methylation markers annotated to transcription start sites (a) or gene bodies (b), split by upregulated, downregulated or neutral status of annotated genes. Up- and down-regulation is assessed here by the significance of the detrended *f*_*up*_ metric with a threshold of *P*_*Bonf*_ < 0.05. (c) Comparison of probes mapping outside of the gene body on GSTM5 against similar probes annotated to all other genes. (d) Comparison of probes mapping specifically to the gene body of NRXN1 against similar probes annotated to all other genes.
(PDF)

S9 Fig Paired tumor-normal expression for ADH genes and ALDH2. (PDF)

S1 Table Fraction upregulated (*f*_*up*_) statistics for genome-wide data. Includes panels for gene, miRNA, and methylation datasets.
(XLSX)

S2 Table Analysis of microarray datasets pulled from GEO. Includes panels describing all 10 microarray datasets obtained from GEO and *f*_*up*_ statistics for all datasets individual and in aggregate.
(XLSX)

S3 Table Gene set enrichment analysis on fraction upregulated statistic. Includes summary panel of non-redundant gene-sets with significant association with *f*_*up*_ as well as a panel listing all gene-sets in the test space.
(XLSX)

S4 Table Association of features with proliferation and detrended *f*_*up*_ scores. Includes panels for gene, miRNA, and methylation datasets.
(XLSX)

S5 Table Gene set enrichment analysis on detrended fraction upregulated statistic. Includes summary panel of non-redundant gene-sets with significant association with *f*_*up*_ as well as a panel listing all gene-sets in the test space.
(XLSX)
