# Supplementary figures and images for: Analysis of Matched Tumor and Normal Profiles Reveals Common Transcriptional and Epigenetic Signals Shared across Cancer Types
Source: PLoS One. 2015 Nov 10;10(11):e0142618. doi: 10.1371/journal.pone.0142618 (PMC4640835; doi:10.1371/journal.pone.0142618)

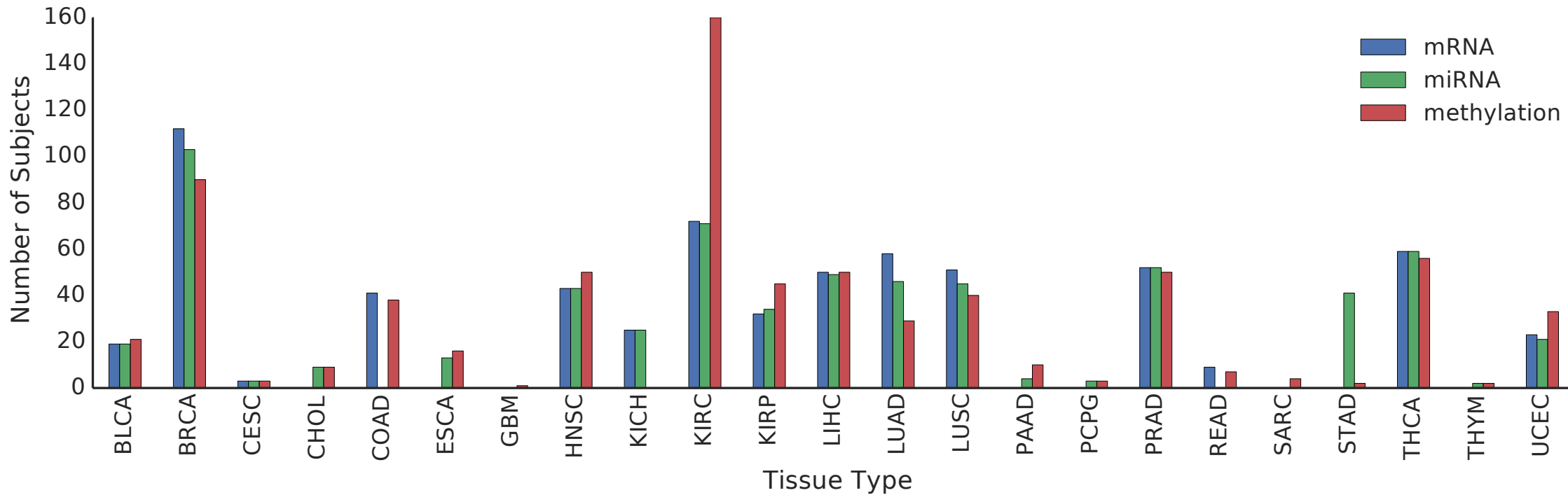

Supplement: S1 Fig — (PDF) [file pone.0142618.s001.pdf]

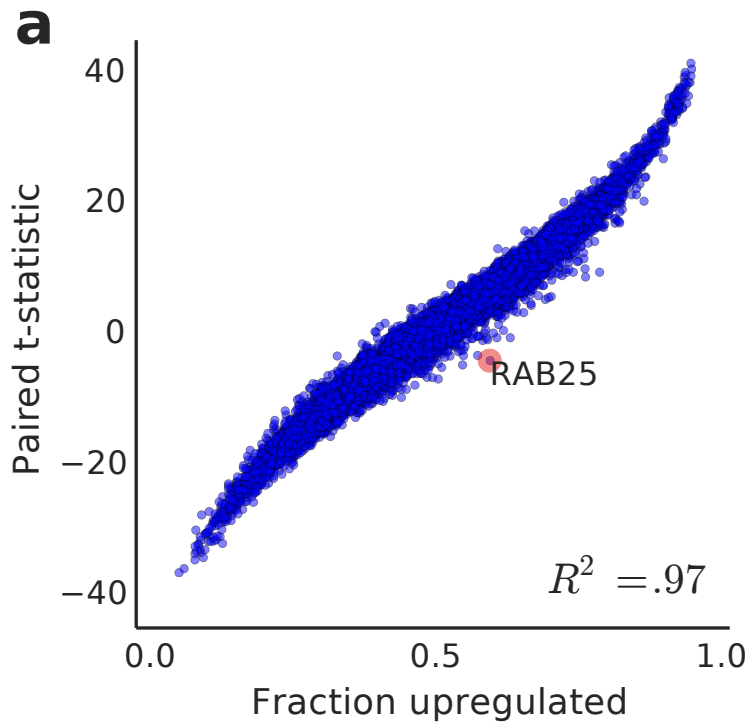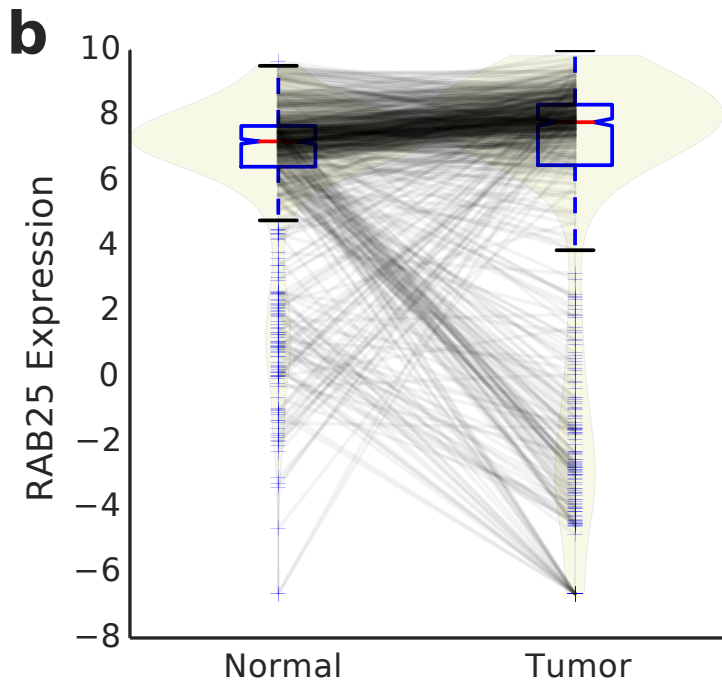

Supplement: S2 Fig — Shown for all genes across the pan-cancer TCGA mRNA sequencing cohort. (PDF) [file pone.0142618.s002.pdf]

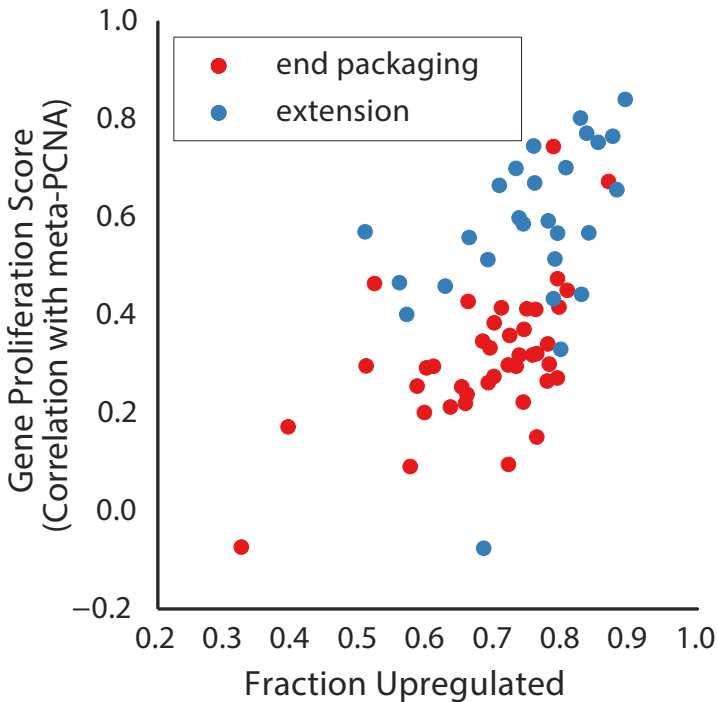

Supplement: S3 Fig — (PDF) [file pone.0142618.s003.pdf]

**a**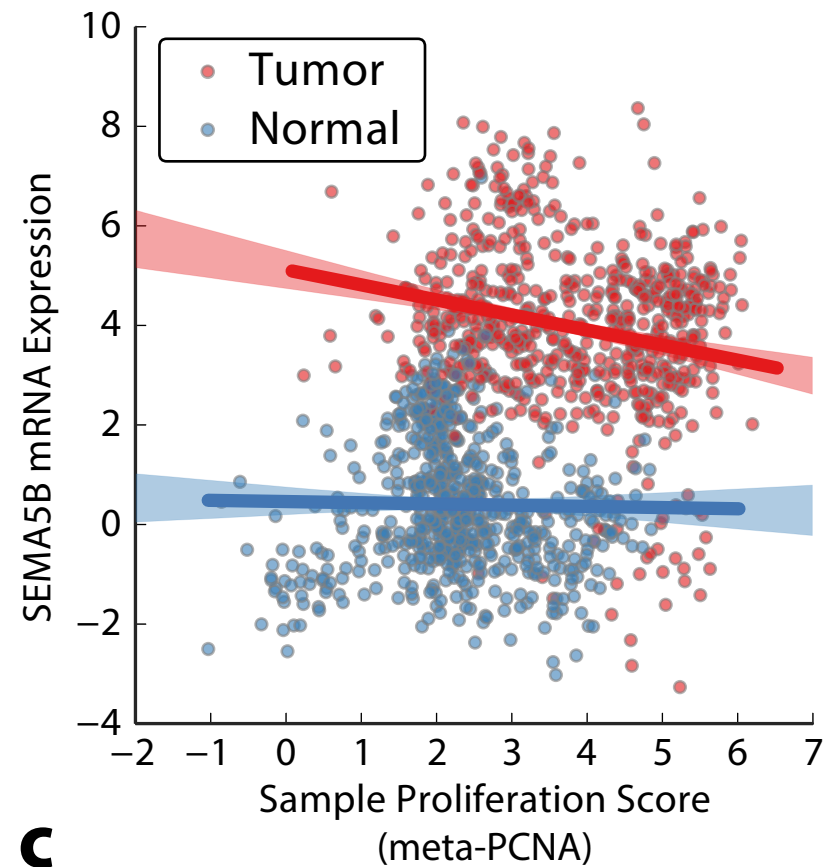**b**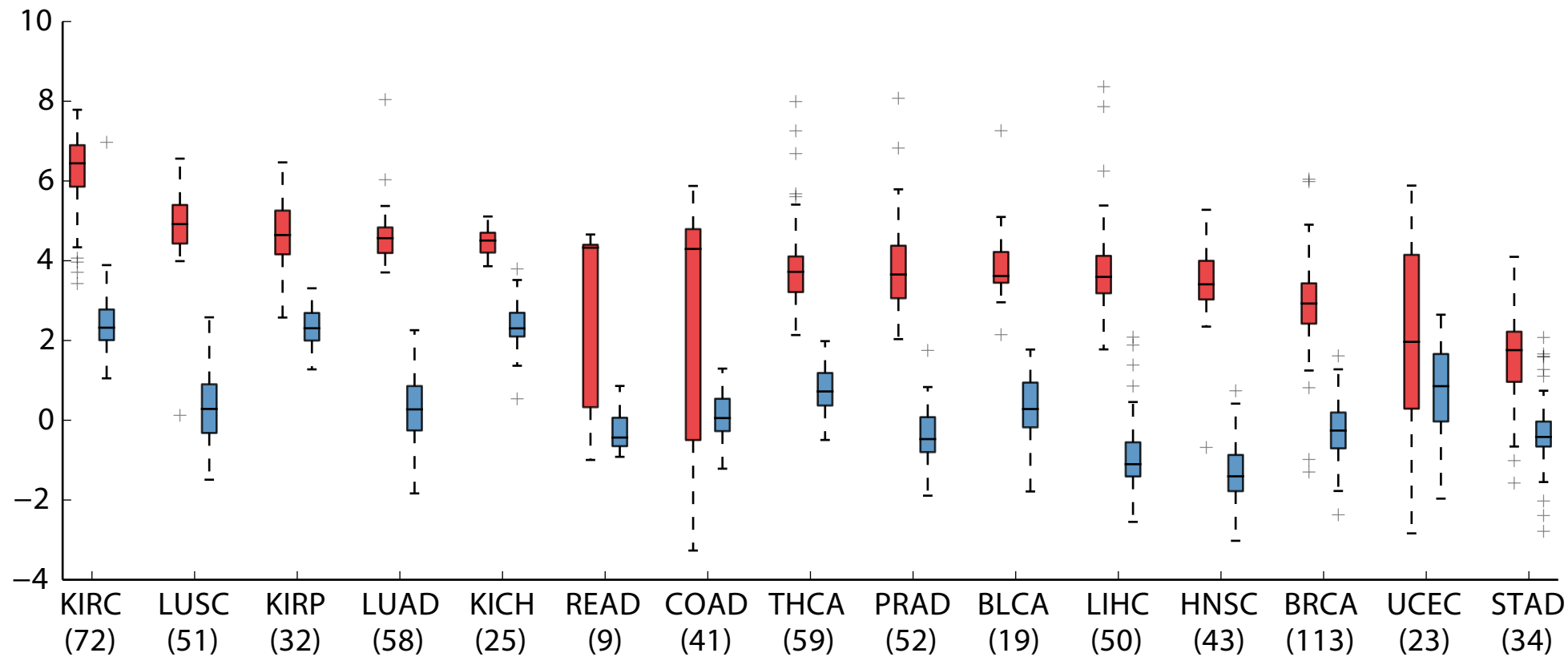**c**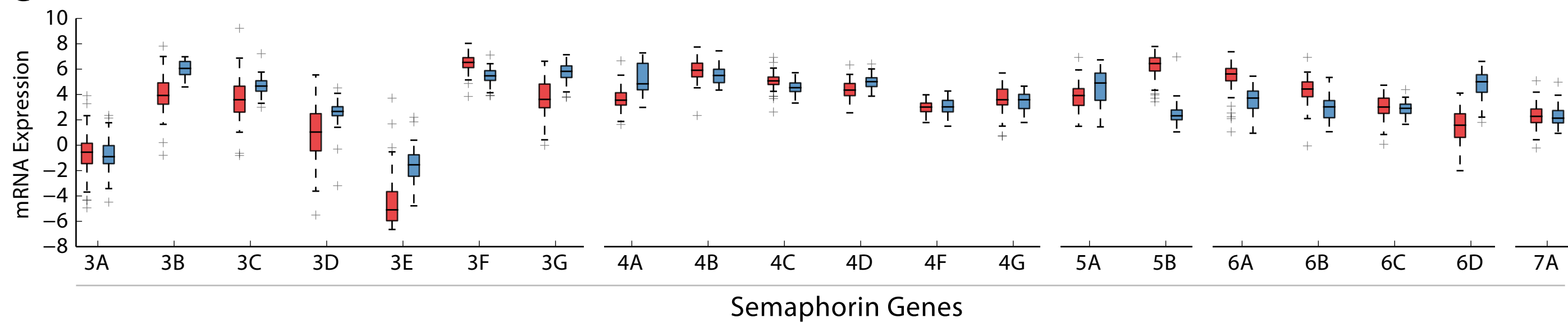

Supplement: S4 Fig — (a) Scatter-plot comparing SEMA5B gene expression profiles to proliferation scores across matched tumor and normal samples. Lines indicate linear regression figs of tumor (red) and normal (blue) samples, shaded regions indicate 95% confidence intervals. (b) Comparison of matched tumor and normal profiles for SEMA5B expression, grouped by tissue type. (c) Comparison of matched tumor and normal profiles for all SEMA protein family of genes in renal cell carcinoma (note that the x-tick labels correspond to the gene suffix, e.g. 3A represents SEMA3A). (PDF) [file pone.0142618.s004.pdf]

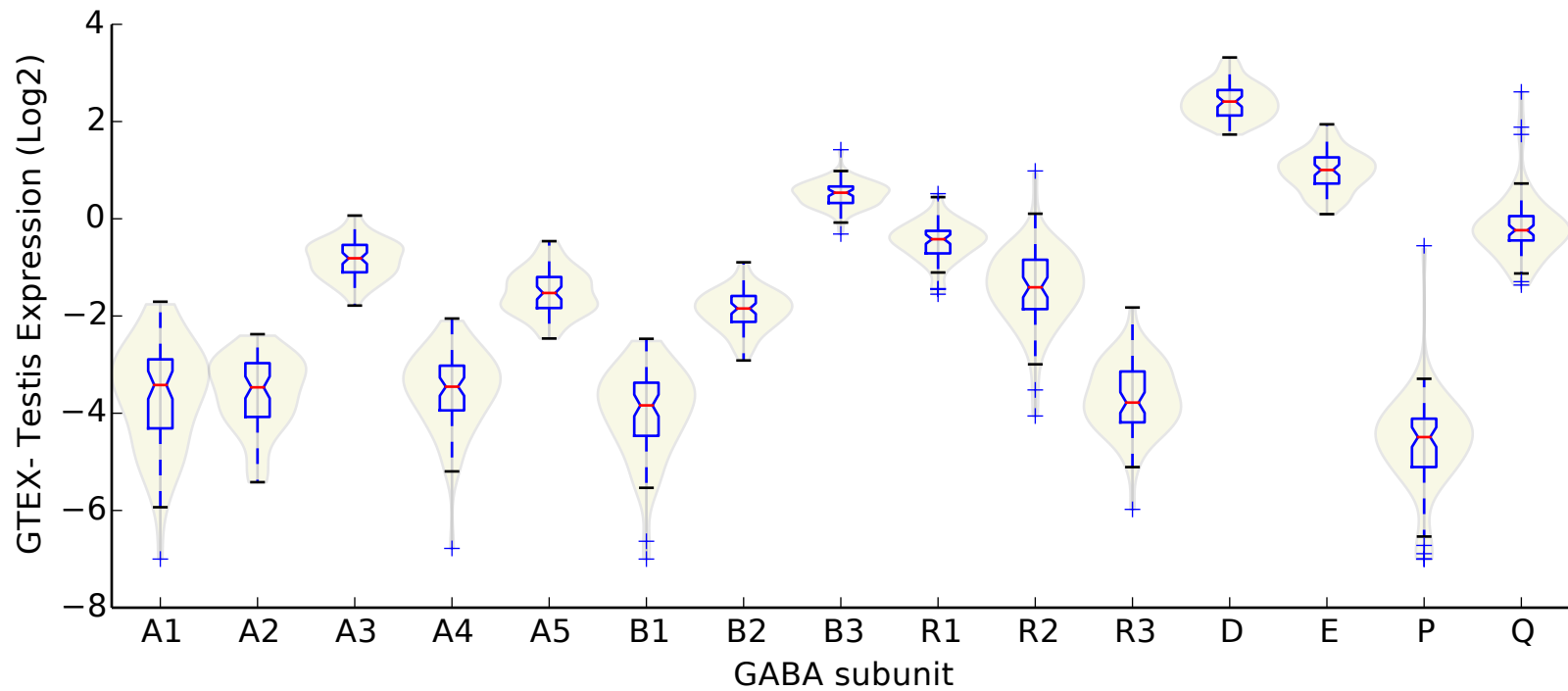

Supplement: S5 Fig — Data obtained from the Genotype-Tissue Expression (GTEX) project [22]. (PDF) [file pone.0142618.s005.pdf]

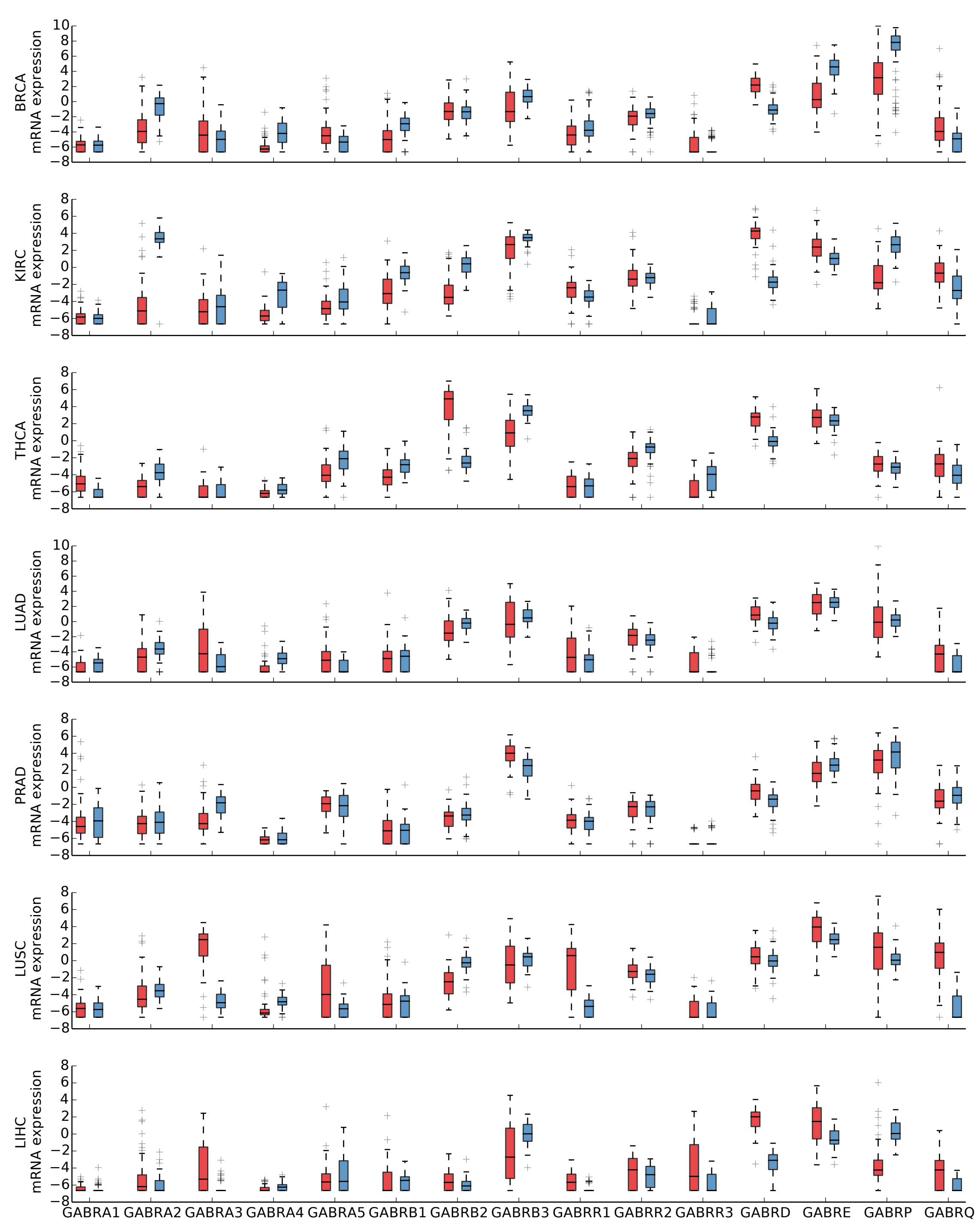

Supplement: S6 Fig — (PDF) [file pone.0142618.s006.pdf]

**a**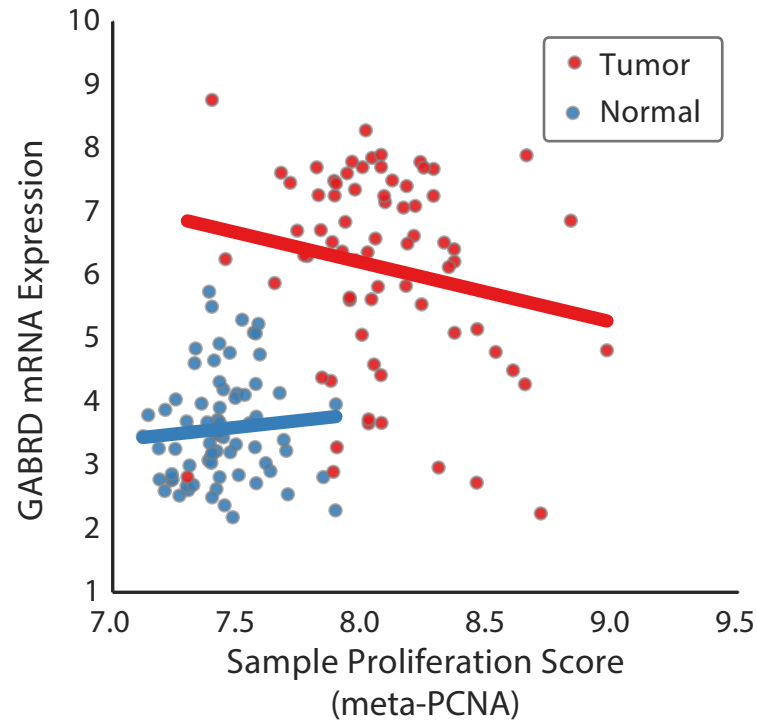**b**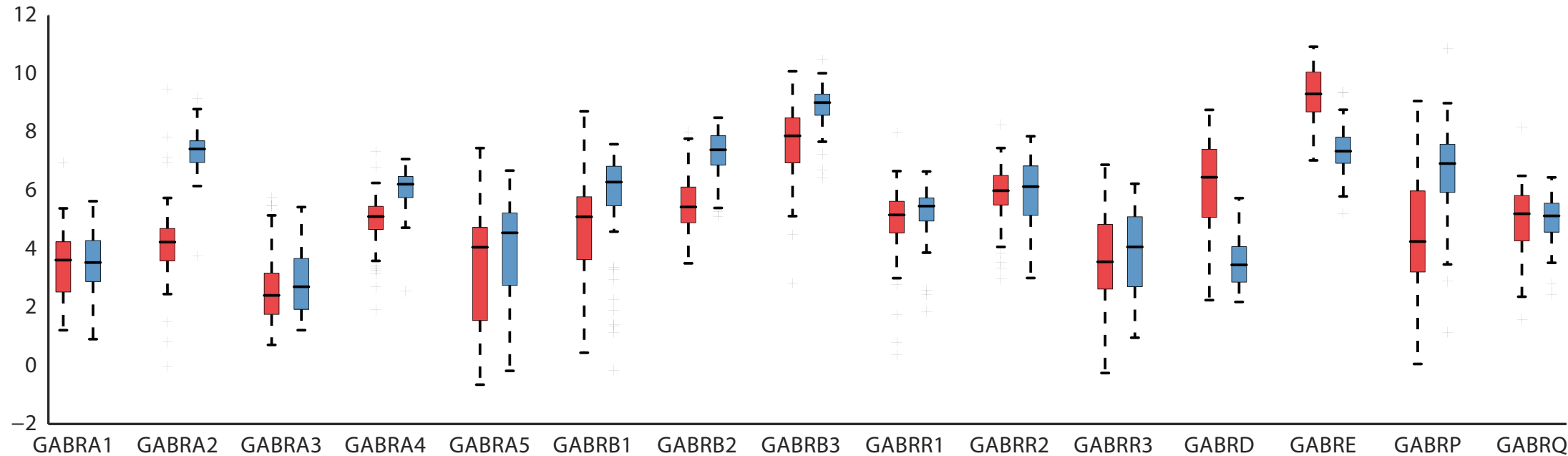

Supplement: S7 Fig — (a) Scatter plot comparing GABRD gene expression profiles to proliferation scores across matched tumor and normal samples. (b) Comparison of matched tumor and normal profiles for all GABA protein subunits. (PDF) [file pone.0142618.s007.pdf]

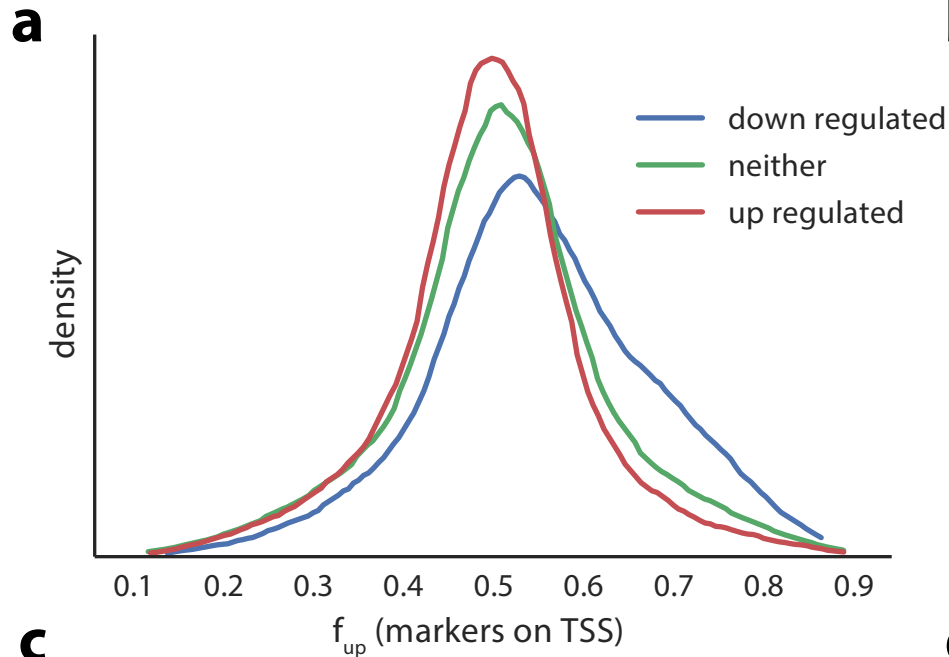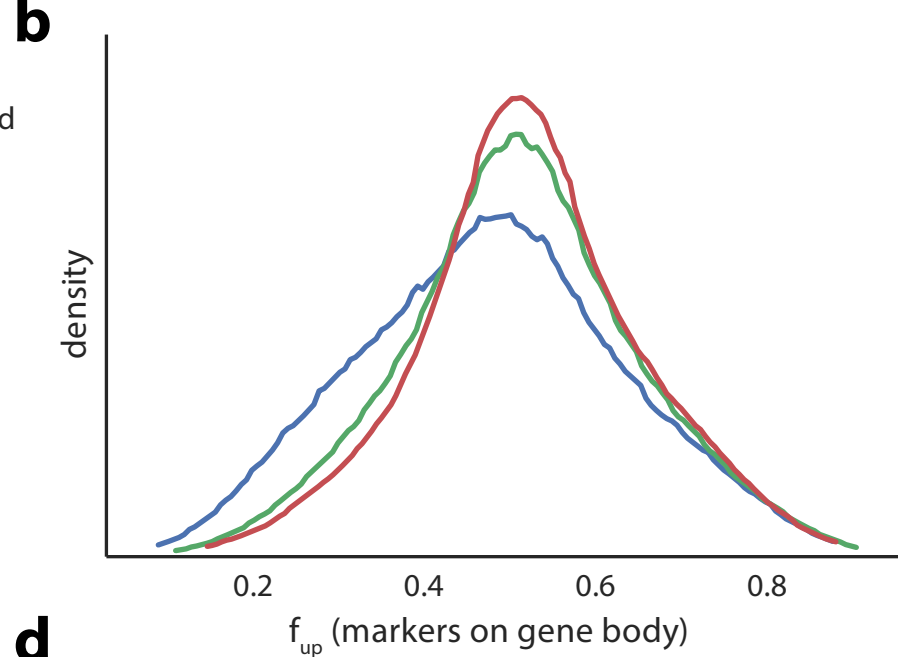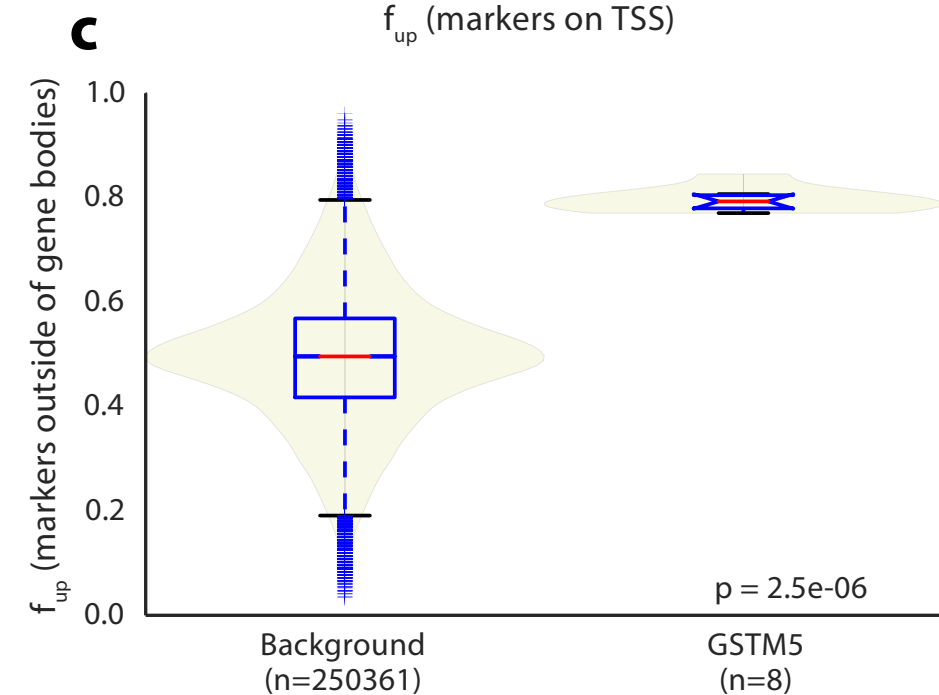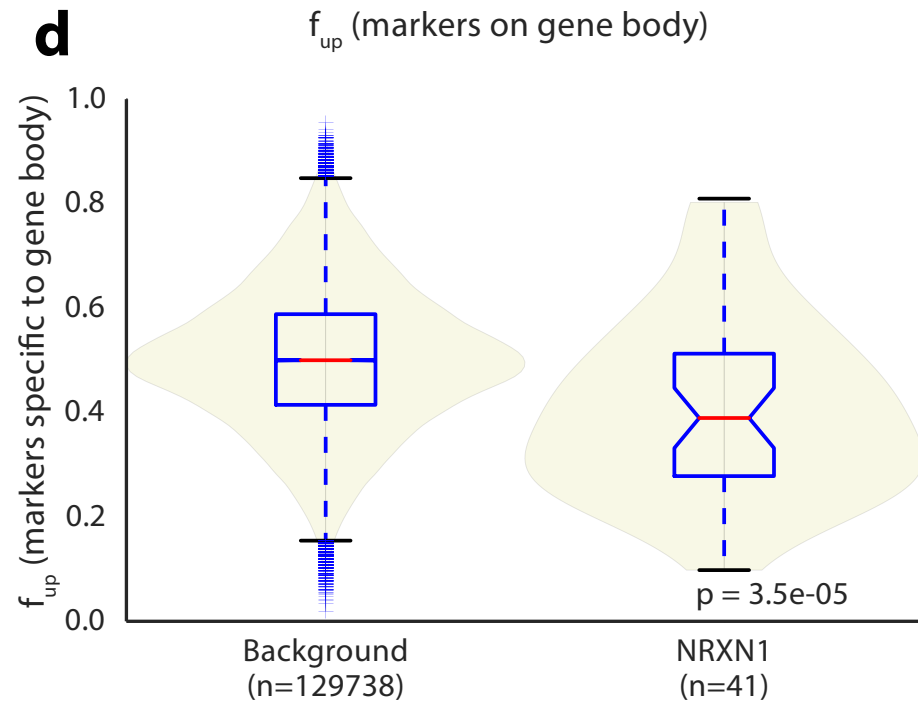

Supplement: S8 Fig — (a-b) Distribution of methylation markers annotated to transcription start sites (a) or gene bodies (b), split by upregulated, downregulated or neutral status of annotated genes. Up- and down-regulation is assessed here by the significance of the detrended f up metric with a threshold of P Bonf < 0.05. (c) Comparison of probes mapping outside of the gene body on GSTM5 against similar probes annotated to all other genes. (d) Comparison of probes mapping specifically to the gene body of NRXN1 against similar probes annotated to all other genes. (PDF) [file pone.0142618.s008.pdf]

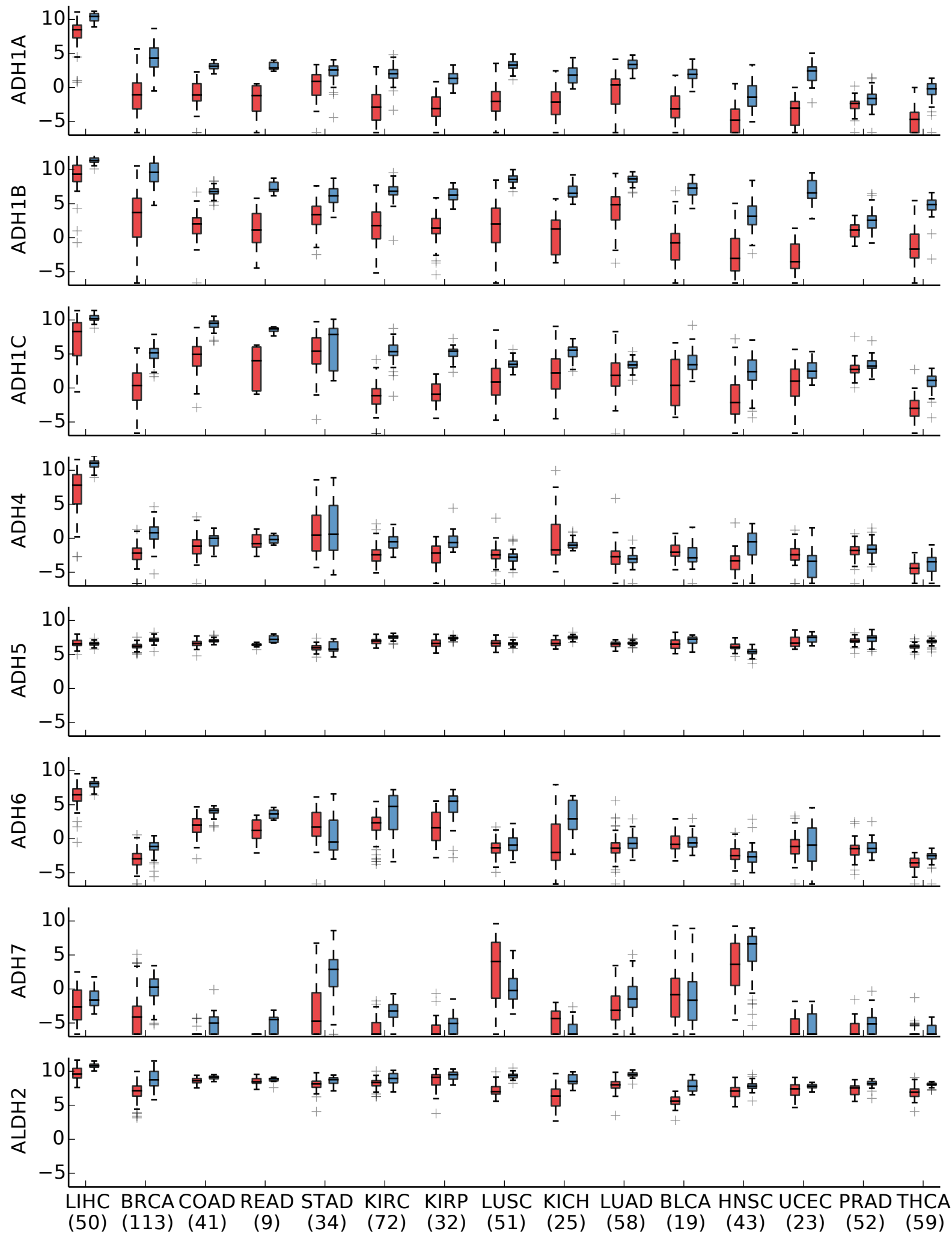

Supplement: S9 Fig — (PDF) [file pone.0142618.s009.pdf]
